# Supplementary material for: Unifying speed limit, thermodynamic uncertainty relation and Heisenberg principle via bulk-boundary correspondence
Source: Nat Commun. 2023 May 17;14:2828. doi: 10.1038/s41467-023-38074-8 (PMC10192383; doi:10.1038/s41467-023-38074-8)
Supplement: Supplementary file 1 — Supplementary Information [file 41467_2023_38074_MOESM1_ESM.pdf]

Supplementary Information for "Unifying Speed Limit, Thermodynamic Uncertainty Relation and Heisenberg Principle via Bulk-Boundary Correspondence"

Yoshihiko Hasegawa[1*]
[1] *Department of Information and Communication Engineering, Graduate School of Information Science and Technology, The University of Tokyo, Tokyo 113-8656, Japan*

SUPPLEMENTARY NOTE 1: CONTINUOUS MATRIX PRODUCT STATE

This subsection explains the calculations associated with the continuous matrix product state. For notational convenience, we define

$$\begin{aligned} Q(t) &\equiv \mathfrak{U}(t; H_{\mathrm{sys}}, \{L_m\}) \\ &= \mathbb{T}\exp\left[-i\int_0^t ds\left\{H_{\mathrm{sys}}\otimes\mathbb{I}_{\mathrm{fld}}+\sum_m\left(iL_m\otimes\phi_m^\dagger(s)-iL_m^\dagger\otimes\phi_m(s)\right)\right\}\right], \end{aligned} \tag{1}$$

where $\mathfrak{U}$ is shown in Eq. (5) in the main text. The continuous matrix product state is given by [Eq. (4) in the main text]

$$|\Phi(t)\rangle = Q(t)\,|\psi(0)\rangle\otimes|\mathrm{vac}\rangle\,. \tag{2}$$

From Eq. (1), we have

$$\begin{aligned} dQ(t) &= Q(t+dt)-Q(t) \\ &= \left[\exp\left[-i\left\{H_{\mathrm{sys}}dt\otimes\mathbb{I}_{\mathrm{fld}}+\sum_m\left(iL_m\otimes d\phi_m^\dagger(t)-iL_m^\dagger\otimes d\phi_m(t)\right)\right\}\right]-1\right]Q(t) \\ &= \sum_{n=1}^{\infty}\frac{1}{n!}\left[-i\left\{H_{\mathrm{sys}}dt\otimes\mathbb{I}_{\mathrm{fld}}+\sum_m\left(iL_m\otimes d\phi_m^\dagger(t)-iL_m^\dagger\otimes d\phi_m(t)\right)\right\}\right]^n Q(t), \end{aligned} \tag{3}$$

where

$$d\phi_m(t) \equiv \int_t^{t+dt}\phi_m(s)ds, \tag{4}$$

$$d\phi_m^\dagger(t) \equiv \int_t^{t+dt}\phi_m^\dagger(s)ds. \tag{5}$$

Because the terms with $n\geq 3$ vanish, Eq. (3) yields

$$d\,|\Phi(t)\rangle = \left[-iH_{\mathrm{sys}}dt\otimes\mathbb{I}_{\mathrm{fld}}+\sum_m L_m\otimes d\phi_m^\dagger(t)-\frac{1}{2}\sum_m L_m^\dagger L_m dt\otimes\mathbb{I}_{\mathrm{fld}}\right]|\Phi(t)\rangle\,, \tag{6}$$

in which the Ito rule is used for $\phi_m(s)$. Then $|\Phi(t)\rangle$ is represented by

$$|\Phi(t)\rangle = \mathbb{T}\exp\left[-i\int_0^t ds\left(H_{\mathrm{eff}}\otimes\mathbb{I}_{\mathrm{fld}}+\sum_m iL_m\otimes\phi_m^\dagger(s)\right)\right]|\psi(0)\rangle\otimes|\mathrm{vac}\rangle\,, \tag{7}$$

* hasegawa@biom.t.u-tokyo.ac.jp

where $H_{\rm eff}$ is the effective Hamiltonian, defined by

$$H_{\rm eff} \equiv H_{\rm sys} - \frac{i}{2}\sum_{m=1}^{M} L_m^\dagger L_m. \tag{8}$$

We now consider an alternative representation of $|\Psi(t)\rangle$. Based on Eq. (7), $|\Psi(\tau)\rangle$ can be expressed by

$$|\Psi(\tau)\rangle = \mathbb{T}\exp\left[-i\int_0^\tau ds \left(H_{\rm eff}\otimes\mathbb{I}_{\rm fld} + \sum_m iL_m\otimes\phi_m^\dagger(s)\right)\right]|\psi(0)\rangle\otimes|{\rm vac}\rangle\,. \tag{9}$$

Let us consider a discretization from 0 to $\Delta s \equiv \tau/N$, where N is a sufficiently large natural number. Then the one-step evolution of Eq. (9) is given by

$$|\Psi(\tau)\rangle_{\Delta s} = \left[(\mathbb{I} - i\Delta s H_{\rm eff}\otimes\mathbb{I}_{\rm fld}) + \sum_m\left(L_m\otimes\Delta\phi_m^\dagger\right)\right]|\psi(0)\rangle\otimes|{\rm vac}\rangle\,. \tag{10}$$

Using $\Delta\phi_m\Delta\phi_{m'}^\dagger = \delta_{mm'}\Delta s$, which can be derived from the canonical commutation relation, we have

$$\begin{aligned}
{\rm Tr}_{\rm fld}\left[|\Psi(\tau)\rangle_{\Delta s}\langle\Psi(\tau)|_{\Delta s}\right] &= (\mathbb{I}_{\rm sys} - i\Delta s H_{\rm eff})|\psi(0)\rangle\langle\psi(0)|\left(\mathbb{I}_{\rm sys} + i\Delta s H_{\rm eff}^\dagger\right) + \sum_m\left(L_m\sqrt{\Delta s}\right)|\psi(0)\rangle\langle\psi(0)|\left(L_m^\dagger\sqrt{\Delta s}\right)\\
&= \sum_{m=0}^{M} V_m|\psi(0)\rangle\langle\psi(0)|V_m^\dagger,
\end{aligned} \tag{11}$$

where V_m comprises the Kraus operators. These are defined by

$$V_0 \equiv \mathbb{I}_{\rm sys} - i\Delta s H_{\rm eff}, \tag{12}$$

$$V_m \equiv \sqrt{\Delta s}L_m \quad (1\le m\le M). \tag{13}$$

Generalizing Eq. (11), it is possible to compute ${\rm Tr}_{\rm fld}\left[|\Psi(t_1)\rangle\langle\Psi(t_2)|\right]$ as

$${\rm Tr}_{\rm fld}\left[|\Psi(t_1)\rangle\langle\Psi(t_2)|\right] = \sum_{m_{N-1}}\cdots\sum_{m_0} V_{m_{N-1}}(t_1)\cdots V_{m_0}(t_1)|\psi(0)\rangle\langle\psi(0)|V_{m_0}^\dagger(t_2)\cdots V_{m_{N-1}}^\dagger(t_2), \tag{14}$$

where $V_m(t)$ are defined by

$$V_0(t) \equiv \mathbb{I}_{\rm sys} - i\Delta s\frac{t}{\tau}H_{\rm eff}, \tag{15}$$

$$V_m(t) \equiv \sqrt{\Delta s}\sqrt{\frac{t}{\tau}}L_m \quad (1\le m\le M). \tag{16}$$

For $N\to\infty$, Eq. (14) yields the two-sided Lindblad equation [cf. Eq. (31)].

SUPPLEMENTARY NOTE 2: CLASSICAL FISHER INFORMATION

In this section, we calculate the classical Fisher information for a classical Markov process. We first consider the general parameter inference in a classical Markov process. Let $\theta\in\mathbb{R}$ be a parameter of interest. Suppose that there are K jump events in a trajectory, and let s_l ($l\in\{0,1,2,\cdots,K\}$) be the time stamp for the lth jump event with $s_0=0$ and $s_{K+1}=\tau$. Let Y_{μ_l} be the state after the lth jump event, where Y_{μ_0} is the initial state of the process and $W_{\mu\nu}(\theta)$ is the transition rate from Y_ν to Y_μ, which depends on the parameter θ, and $P(\mu_0;\theta)$ is the initial distribution of the Markov process, which is also dependent on θ. From the path integral representation [1], the probability of the trajectory Γ with the initial state Y_{μ_0} is

$$\mathcal{P}(\mu_0,\Gamma;\theta) = P(\mu_0;\theta)\mathcal{P}(\Gamma|\mu_0;\theta), \tag{17}$$

where $\mathcal{P}(\Gamma|\mu_0;\theta)$ is the conditional probability of Γ given the initial state Y_{μ_0}. $\mathcal{P}(\Gamma|\mu_0;\theta)$ is given by

$$\ln\mathcal{P}(\Gamma|\mu_0;\theta) = \sum_{l=1}^{K}\ln W_{\mu_l\mu_{l-1}}(\theta) - \sum_{l=0}^{K}\int_{s_l}^{s_{l+1}} ds\, R(\mu_l;\theta), \tag{18}$$

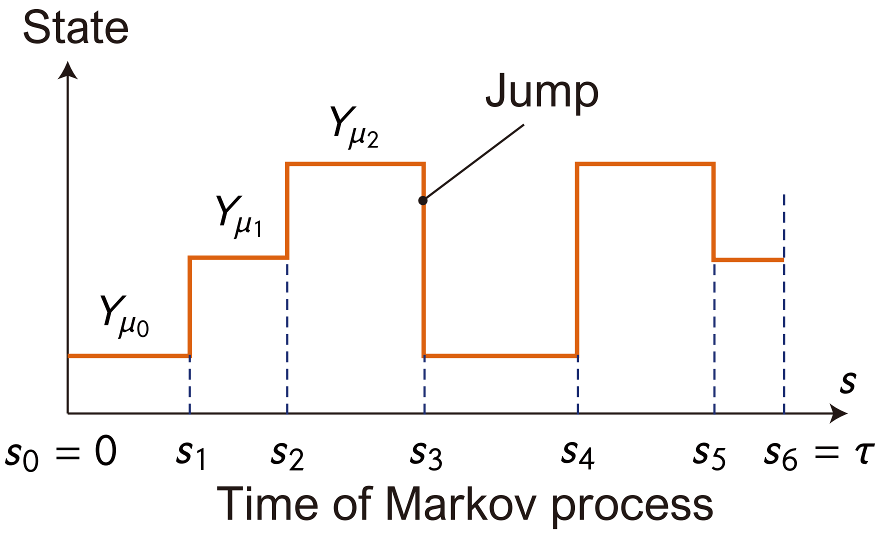

FIG. S1. **Example of a classical Markov process.** A diagram of a process that starts from $s = 0$ and ends at $s = \tau$ while undergoing $K = 5$ jump events. Here, s_l denotes the time stamp for the lth jump event, where $s_0 \equiv 0$ and $s_{K+1} = s_6 = \tau$. In addition, Y_{μ_l} denotes the state after the lth jump event.

where $R(\mu;\theta)$ is the escape rate defined by

$$R(\mu;\theta) \equiv \sum_{\nu(\neq\mu)} W_{\nu\mu}(\theta). \tag{19}$$

Using Eq. (18), we can evaluate the Fisher information:

$$-\left\langle \frac{\partial^2}{\partial\theta^2} \ln \mathcal{P}(\mu_0,\Gamma;\theta) \right\rangle = -\left\langle \frac{\partial^2}{\partial\theta^2} \ln P(\mu_0;\theta) \right\rangle - \left\langle \sum_{l=1}^{K} \frac{\partial^2}{\partial\theta^2} \ln W_{\mu_l\mu_{l-1}}(\theta) \right\rangle + \left\langle \sum_{l=0}^{K} \int_{s_l}^{s_{l+1}} ds\, \frac{\partial^2}{\partial\theta^2} R(\mu_l;\theta) \right\rangle, \tag{20}$$

where the bracket denotes the expectation. The second term on the right hand side of Eq. (20) becomes

$$\begin{aligned}\left\langle \sum_{l=1}^{K} \frac{\partial^2}{\partial\theta^2} \ln W_{\mu_l\mu_{l-1}}(\theta) \right\rangle &= \int_0^\tau ds \sum_{\mu,\nu,\mu\neq\nu} P(\mu,s;\theta) W_{\nu\mu}(\theta) \frac{\partial^2}{\partial\theta^2} \ln W_{\nu\mu}(\theta) \\ &= \int_0^\tau ds \sum_{\mu,\nu,\mu\neq\nu} P(\mu,s;\theta) W_{\nu\mu}(\theta) \left[-\frac{(\partial_\theta W_{\nu\mu}(\theta))^2}{W_{\nu\mu}(\theta)^2} + \frac{\partial_\theta^2 W_{\nu\mu}(\theta)}{W_{\nu\mu}(\theta)} \right] \\ &= \int_0^\tau ds \sum_{\mu,\nu,\mu\neq\nu} P(\mu,s;\theta) \left[-\frac{(\partial_\theta W_{\nu\mu}(\theta))^2}{W_{\nu\mu}(\theta)} + \frac{\partial^2}{\partial\theta^2} W_{\nu\mu}(\theta) \right].\end{aligned} \tag{21}$$

Similarly, the third term on the right hand side of Eq. (20) becomes

$$\left\langle \sum_{l=0}^{K} \int_{s_l}^{s_{l+1}} ds\, \frac{\partial^2}{\partial\theta^2} R(\mu_l;\theta) \right\rangle = \int_0^\tau ds \sum_{\mu,\nu,\mu\neq\nu} P(\mu,s;\theta) \frac{\partial^2}{\partial\theta^2} W_{\nu\mu}(\theta). \tag{22}$$

Combining Eqs. (21) and (22), the Fisher information is

$$\mathcal{I}(\theta) = -\left\langle \frac{\partial^2}{\partial\theta^2} \ln P(\mu_0;\theta) \right\rangle + \int_0^\tau ds \sum_{\mu,\nu,\mu\neq\nu} P(\mu,s;\theta) \frac{(\partial_\theta W_{\nu\mu}(\theta))^2}{W_{\nu\mu}(\theta)}. \tag{23}$$

In the main text, it is demonstrated that the classical Fisher information [Eq. (19) in the main text] is given by

$$\mathcal{I}(t) = \sum_{\Gamma,\nu} \mathcal{P}(\Gamma,\nu,t) \left(-\frac{\partial^2}{\partial t^2} \ln \mathcal{P}(\Gamma,\nu,t) \right), \tag{24}$$

where $\mathcal{P}(\Gamma,\nu,t)$ is the probability of measuring a trajectory Γ and Y_ν at the end time. Knowing a specific trajectory Γ and the final state Y_ν, we can uniquely specify the state change of the dynamics. Therefore, Eq. (24) can be rewritten as

$$\mathcal{I}(t)=\sum_{\mu_0,\Gamma}\mathcal{P}(\mu_0,\Gamma,t)\left(-\frac{\partial^2}{\partial t^2}\ln\mathcal{P}(\mu_0,\Gamma,t)\right), \tag{25}$$

where $\mathcal{P}(\mu_0,\Gamma,t)$ is the probability of measuring a trajectory Γ given the initial state Y_{μ_0}. Equations (17)–(23) can then be used to calculate the Fisher information. Let $P(\nu,s;\boldsymbol{W})$ be the probability of being Y_ν at time s, the dynamics of which is governed by a classical Markov process with the transition rate $\boldsymbol{W}\equiv\{W_{\mu\nu}\}_{\mu,\nu}$. From Eq. (23), we obtain

$$\begin{aligned}\mathcal{I}(t)&=\int_0^\tau ds\sum_{\nu,\mu,\nu\neq\mu}\frac{P(\nu,s;\frac{t}{\tau}\boldsymbol{W})}{\frac{t}{\tau}W_{\mu\nu}}\left(\frac{\partial\frac{t}{\tau}W_{\mu\nu}}{\partial t}\right)^2\\&=\int_0^\tau ds\sum_{\nu,\mu,\nu\neq\mu}\frac{1}{t\tau}P\left(\nu,s;\frac{t}{\tau}\boldsymbol{W}\right)W_{\mu\nu}.\end{aligned} \tag{26}$$

In Eq. (26), the boundary term $P\left(\nu,s=0;\frac{t}{\tau}\boldsymbol{W}\right)$ does not come into play since the initial state does not depend on t. We define the dynamical activity from 0 to t as follows:

$$\mathcal{A}(t;\boldsymbol{W})\equiv\int_0^t ds\sum_{\nu,\mu,\nu\neq\mu}P(\mu,s;\boldsymbol{W})W_{\nu\mu}, \tag{27}$$

which quantifies the average number of jumps in $[0,t]$. Since the transition rate is time-independent, we have the relation:

$$P\left(\nu,s;\frac{t}{\tau}\boldsymbol{W}\right)=P\left(\nu,\frac{t}{\tau}s;\boldsymbol{W}\right). \tag{28}$$

Substituting Eq. (28) into Eq. (26), we obtain

$$\mathcal{I}(t)=\frac{\mathcal{A}\left(\tau;\frac{t}{\tau}\boldsymbol{W}\right)}{t^2}=\frac{\mathcal{A}(t;\boldsymbol{W})}{t^2}, \tag{29}$$

which is Eq. (22) in the main text.

SUPPLEMENTARY NOTE 3: QUANTUM FISHER INFORMATION

The geometric bound given by Eq. (34) in the main text concerns the calculation of the quantum Fisher information $\mathcal{J}(t)$, which is expressed by Eq. (35) in the main text. Specifically, we have

$$\langle\Psi(t_2)|\Psi(t_1)\rangle=\mathrm{Tr}_{\mathrm{sys,fld}}\left[|\Psi(t_1)\rangle\langle\Psi(t_2)|\right]=\mathrm{Tr}_{\mathrm{sys}}\left[\zeta(\tau;t_1,t_2)\right], \tag{30}$$

where $\zeta(\tau;t_1,t_2)\equiv\mathrm{Tr}_{\mathrm{fld}}\left[|\Psi(t_1)\rangle\langle\Psi(t_2)|\right]$. From Eq. (14), $\zeta(s;t_1,t_2)$ obeys the two-sided Lindblad equation [2]:

$$\frac{d}{ds}\zeta(s;t_1,t_2)=-iH_{1,\mathrm{sys}}\zeta+i\zeta H_{2,\mathrm{sys}}+\sum_m L_{1,m}\zeta L_{2,m}^\dagger-\frac{1}{2}\sum_m\left[L_{1,m}^\dagger L_{1,m}\zeta+\zeta L_{2,m}^\dagger L_{2,m}\right], \tag{31}$$

where $H_{1,\mathrm{sys}}\equiv(t_1/\tau)H_{\mathrm{sys}}$ and $L_{1,m}\equiv\sqrt{t_1/\tau}L_m$ ($H_{2,\mathrm{sys}}$ and $L_{2,m}$ are defined in a similar manner). Note that $\zeta(s;t_1,t_2)$ is not a density operator because $\mathrm{Tr}_{\mathrm{sys}}[\zeta(s;t_1,t_2)]\neq 1$ in general. Calculating Eq. (31) with the initial state $\zeta(0;t_1,t_2)=|\psi(0)\rangle\langle\psi(0)|$ to $s=\tau$, the fidelity can be computed. Practically, we compute $\mathcal{J}(t)$ based on the relationship:

$$\mathcal{J}(t)=\frac{8}{dt^2}\left[1-|\langle\Psi(t)|\Psi(t+dt)\rangle|\right], \tag{32}$$

where dt is taken to be a sufficiently small increment. Equation (32) can be shown by using $|\partial_t\Psi(t)\rangle\,dt=|\Psi(t+dt)\rangle-|\Psi(t)\rangle$. Since $|\Psi(t)\rangle$ can be calculated by Eq. (31), we can calculate the quantum Fisher information via Eq. (32).

SUPPLEMENTARY NOTE 4: INITIALLY MIXED STATE CASE

The calculation in the main text assumes an initially pure state, i.e., $\rho(0) = |\psi(0)\rangle \langle\psi(0)|$ but here we consider an initially mixed-state case. We can introduce an ancilla that purifies the initial state:

$$\rho(0) = \mathrm{Tr}_{\mathrm{anc}}[|\tilde{\psi}(0)\rangle \langle\tilde{\psi}(0)|], \tag{33}$$

where $|\tilde{\psi}(0)\rangle$ is a purification of $\rho(0)$ and $\mathrm{Tr}_{\mathrm{anc}}$ is the trace operation with respect to the ancilla. We define the scaled continuous matrix product state for the purified state as follows:

$$|\tilde{\Psi}(t)\rangle = \tilde{\mathfrak{U}}\left(\tau; \frac{t}{\tau}H_{\mathrm{sys}}, \left\{\sqrt{\frac{t}{\tau}}L_m\right\}\right)|\tilde{\psi}(0)\rangle \otimes |\mathrm{vac}\rangle\,, \tag{34}$$

$$\tilde{\mathfrak{U}}(\tau; H_{\mathrm{sys}}, \{L_m\}) \equiv \mathbb{T}\exp\left[-i\int_0^\tau ds\left(H_{\mathrm{eff}} \otimes \mathbb{I}_{\mathrm{anc}} \otimes \mathbb{I}_{\mathrm{fld}} + \sum_m iL_m \otimes \mathbb{I}_{\mathrm{anc}} \otimes \phi_m^\dagger(s)\right)\right],$$

where $\mathbb{I}_{\mathrm{anc}}$ is the identity operator in the ancilla. In Eq. (34) in the main text, we consider the relation:

$$\frac{1}{2}\int_{t_1}^{t_2} dt\,\sqrt{\mathcal{J}(t)} \geq \arccos\left[|\langle\Psi(t_2)|\Psi(t_1)\rangle|\right]. \tag{35}$$

Using the purified state $|\tilde{\Psi}(t)\rangle$ in Eq. (34), from Eq. (35), the following relation holds:

$$\frac{1}{2}\int_{t_1}^{t_2} dt\,\sqrt{\tilde{\mathcal{J}}(t)} \geq \arccos\left[\left|\langle\tilde{\Psi}(t_2)|\tilde{\Psi}(t_1)\rangle\right|\right], \tag{36}$$

where $\tilde{\mathcal{J}}(t)$ is the quantum Fisher information defined using the purified state:

$$\tilde{\mathcal{J}}(t) \equiv 4\left[\langle\partial_t\tilde{\Psi}(t)|\partial_t\tilde{\Psi}(t)\rangle - \left|\langle\partial_t\tilde{\Psi}(t)|\tilde{\Psi}(t)\rangle\right|^2\right]. \tag{37}$$

Due to the monotonicity of the quantum fidelity, the right hand side of Eq. (36) can be evaluated in the same manner as in the case of the initially pure state [cf. Eq. (44) in the main text]. That is

$$\mathrm{Fid}(\rho(t_1), \rho(t_2)) \geq \mathrm{Fid}(|\tilde{\Psi}(t_1)\rangle, |\tilde{\Psi}(t_2)\rangle), \tag{38}$$

which indicates that Eq. (39) in the main text should hold for the initially mixed state case. Similarly, Eq. (49) in the main text is satisfied for the initially mixed state case.

Next, we consider the evaluation of $\tilde{\mathcal{J}}(t)$, which appears on the left hand side of Eq. (36) and can be computed through the fidelity $\langle\Psi(\tilde{t}_2)|\Psi(\tilde{t}_1)\rangle$. Let us define $\tilde{V}_m$ as follows:

$$\tilde{V}_m(t) \equiv V_m(t) \otimes \mathbb{I}_{\mathrm{anc}} \quad (0 \leq m \leq M), \tag{39}$$

where $V_m(t)$ is defined by Eqs. (15) and (16). We thus obtain

$$\begin{aligned}
\langle\tilde{\Psi}(t_2)|\tilde{\Psi}(t_1)\rangle &= \mathrm{Tr}_{\mathrm{sys,anc,fld}}\left[|\tilde{\Psi}(t_1)\rangle\langle\tilde{\Psi}(t_2)|\right] \\
&= \mathrm{Tr}_{\mathrm{sys,anc}}\left[\sum_{m_{N-1}}\cdots\sum_{m_0}\tilde{V}_{m_{N-1}}(t_1)\cdots\tilde{V}_{m_0}(t_1)|\tilde{\psi}(0)\rangle\langle\tilde{\psi}(0)|\tilde{V}_{m_0}^\dagger(t_2)\cdots\tilde{V}_{m_{N-1}}^\dagger(t_2)\right] \\
&= \mathrm{Tr}_{\mathrm{sys}}\left[\sum_{m_{N-1}}\cdots\sum_{m_0}V_{m_{N-1}}(t_1)\cdots V_{m_0}(t_1)\rho(0)V_{m_0}^\dagger(t_2)\cdots V_{m_{N-1}}^\dagger(t_2)\right].
\end{aligned} \tag{40}$$

For $N \to \infty$, the last line of Eq. (40) gives the two-sided Lindblad equation of Eq. (31) with the initial density $\zeta(s = 0; t_1, t_2) = \rho(0)$. Then, when we compute the quantum Fisher information using the two-sided Lindblad equation, Eqs. (39) and (40) in the main text hold for the initially mixed state case.

SUPPLEMENTARY NOTE 5: NUMERICAL SIMULATION

To better understand the bounds obtained from the present calculations, we consider paradigmatic classical and quantum models. For classical dynamics, we consider N_S states Markov process, the dynamics of which is governed by Eq. (16) in the main text. When calculating fluctuations of the observable $\mathcal{C}$ in the classical thermodynamic uncertainty relation, each trajectory is generated by the Gillespie algorithm [3]. The dynamical activity $\mathcal{A}(t)$ is evaluated by performing the numerical integration to Eq. (27), where the time-dependent probability distribution $P(\mu, s; \boldsymbol{W})$ is obtained by the matrix exponential.

For quantum dynamics, we employ a two-level atom driven by a classical laser field. This dynamics is governed by the Lindblad equation [Eq. (1) in the main text], where the Hamiltonian and the jump operator are given by

$$H_{\mathrm{sys}} = \Delta \left|\epsilon_e\right\rangle \left\langle\epsilon_e\right| + \frac{\Omega}{2} \left(\left|\epsilon_e\right\rangle \left\langle\epsilon_g\right| + \left|\epsilon_g\right\rangle \left\langle\epsilon_e\right|\right), \tag{41}$$

$$L = \sqrt{\kappa} \left|\epsilon_g\right\rangle \left\langle\epsilon_e\right|. \tag{42}$$

Here, $|\epsilon_e\rangle$ and $|\epsilon_g\rangle$ denote the excited and ground states, respectively, Δ is the extent of detuning between the laser field and the atomic transition frequencies, Ω is the Rabi-oscillation frequency and κ is the decay rate. The jump operator L induces a jump from the excited state $|\epsilon_e\rangle$ to the ground state $|\epsilon_g\rangle$. To calculate the fluctuation of the observable $\mathcal{C}$, we generate quantum trajectories. Each trajectory obeys the stochastic Schrödinger equation:

$$d\rho = -i[H_{\mathrm{sys}}, \rho]ds + \rho \mathrm{Tr}_{\mathrm{sys}}\left[L\rho L^\dagger\right] ds - \frac{\left\{L^\dagger L, \rho\right\}}{2} ds + \left(\frac{L\rho L^\dagger}{\mathrm{Tr}_{\mathrm{sys}}[L\rho L^\dagger]} - \rho\right) d\mathfrak{n}, \tag{43}$$

where $d\mathfrak{n}$ is a noise increment having a value of 1 when a jump event (photon) is detected within ds and otherwise has a value of 0. The conditional expectation of $d\mathfrak{n}$ is $\mathrm{Tr}_{\mathrm{sys}}[L\rho(s)L^\dagger]ds$, where $\rho(s)$ is a solution of Eq. (43).

Speed limit relations

Here, we first calculate the classical speed limit relation. We use a two-state Markov process ($N_S = 2$) and calculate $(1/2)\int_0^s \sqrt{\mathcal{A}(t)}/t\, dt$ and $\mathcal{L}_P(P(\nu,0), P(\nu,s))$ in Eq. (24) in the main text. These are shown by the dashed and solid lines, respectively, in Figs. S2(a) and (b). Figures S2(a) and (b) employ different initial distributions, (a) $[P(\nu,0)]_{\nu=1,2} = [1,0]$ and (b) $[P(\nu,0)]_{\nu=1,2} = [0.6, 0.4]$, while the other settings are the same. From Fig. S2(a), it is evident that $(1/2)\int_0^s \sqrt{\mathcal{A}(t)}/t\, dt$ and $\mathcal{L}_P(P(\nu,0), P(\nu,s))$ are almost equivalent for $s < 0.5$. The classical speed limit concerns two inequalities. Since the first inequality is Eq. (18) in the main text and saturates when the dynamics is the geodesic with respect to the Fisher information metric, this inequality saturates when s is sufficiently small. The second inequality is Eq. (43) in the main text, which concerns the monotonicity with respect to stochastic maps. For Fig. S2(b), the difference between $(1/2)\int_0^s \sqrt{\mathcal{A}(t)}/t\, dt$ and $\mathcal{L}_P(P(\nu,0), P(\nu,s))$ is large even at an earlier time. Therefore, the divergence in Fig. S2(b) is caused by the second inequality. This result indicates that, if the initial distribution is $[P(\nu,0)]_{\nu=1,2} = [0.6, 0.4]$, there is greater ambiguity between the trajectory information and the distribution $P(\nu,s)$.

We now consider the quantum case. We use the two-level atom model to calculate $(1/2)\int_0^s \sqrt{\mathcal{B}(t)}/t\, dt$ and $\mathcal{L}_D(\rho(0), \rho(s))$ in Eq. (39) in the main text, as indicated by the dashed and solid lines, respectively, in Figs. S2(c) and (d). Here, we consider two cases (c) $\kappa = 0$ and (d) $\kappa = 2$ while the other settings are the same. For $\kappa = 0$, the jump operator L vanishes and hence the system reduces to a closed quantum dynamics, meaning that Figs. S2(c) and (d) highlight the differences between closed and open quantum dynamics. It is evident that, in both cases, $(1/2)\int_0^s \sqrt{\mathcal{B}(t)}/t\, dt$ is bounded from below by $\mathcal{L}_D(\rho(0), \rho(s))$ and the extent of saturation is greater for lower values of s, which is similar to the classical case. Comparing Figs. S2(c) and (d) shows that $(1/2)\int_0^s \sqrt{\mathcal{B}(t)}/t\, dt$ and $\mathcal{L}_D(\rho(0), \rho(s))$ are closer for (c), suggesting that Eq. (39) in the main text is tighter for the closed dynamics.

Thermodynamic uncertainty relations

We next focus on the classical thermodynamic uncertainty relations. We use a classical Markov process with N_S states, where N_S is determined at random. After determining N_S, we decide the topology of the Markov process. The other model parameters, including the transition rate $W_{\mu\nu}$, are randomly selected (see the caption of Fig. S3 for the parameter ranges). Moreover, we randomly generate the initial distribution $P(\nu, 0)$. For each selected parameter

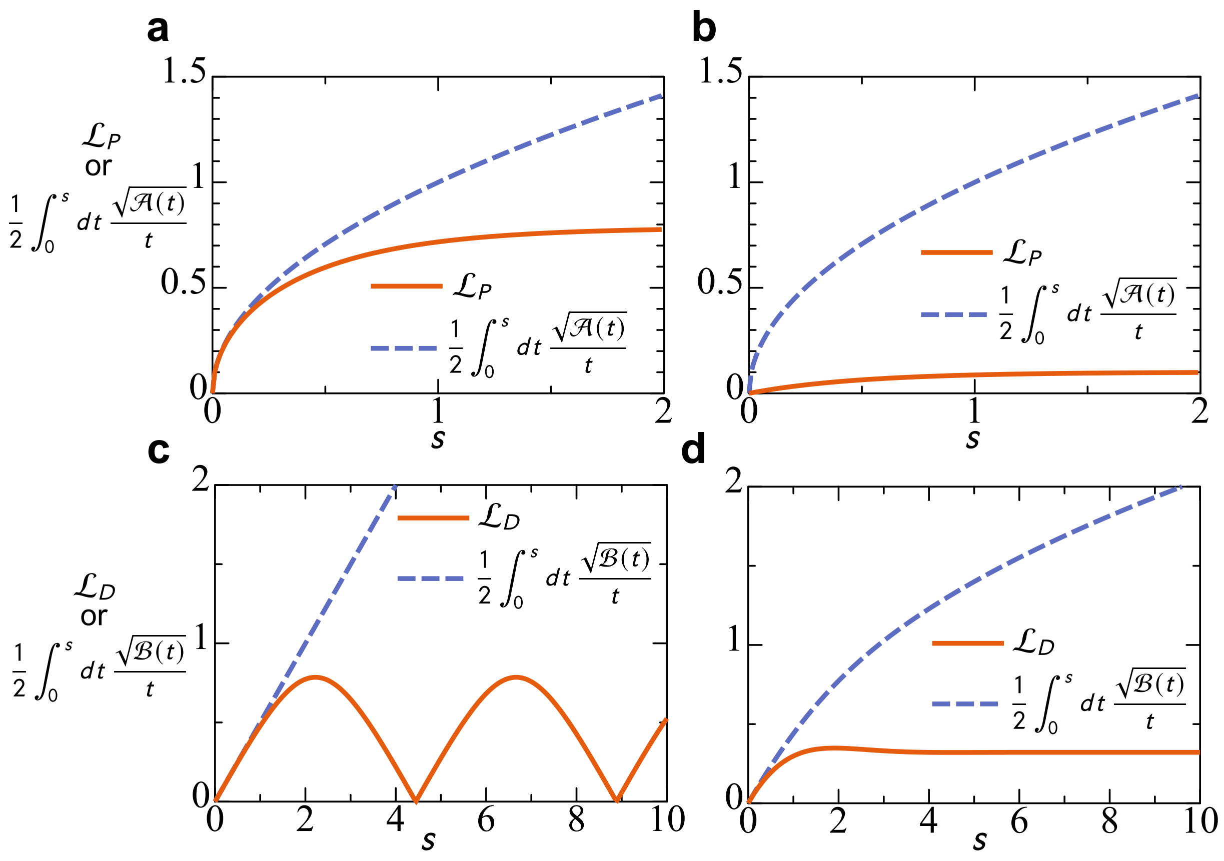

FIG. S2. **Numerical calculations of the classical and quantum speed limit relations.** **a** and **b** Classical speed limit relation for the Markov process ($N_S = 2$). $(1/2)\int_0^s \sqrt{\mathcal{A}(t)}/t\,dt$ and $\mathcal{L}_P(P(\nu,0),P(\nu,s))$ are plotted with the dashed and solid lines, respectively as functions of time s for different initial probabilities: **a** $[P(\nu,0)]_{\nu=1,2} = [1,0]$ and **b** $[P(\nu,0)]_{\nu=1,2} = [0.6,0.4]$. The transition matrix W is set to $W_{12} = W_{21} = 1$. **c** and **d** Quantum speed limit relation for the two-level atom model. $(1/2)\int_0^s \sqrt{\mathcal{B}(t)}/t\,dt$ and $\mathcal{L}_D(\rho(0),\rho(s))$ are plotted with the dashed and solid lines, respectively, as functions of time s for different κ values: **c** $\kappa = 0$, corresponding to the closed quantum dynamics, and **d** $\kappa = 2$. The other parameters are $\Delta = 1$ and $\Omega = 1$.

set, we generate trajectories and calculate $[\![\mathcal{C}]\!]_\tau^2/\langle\mathcal{C}\rangle_\tau^2$. Since we select the initial distribution randomly, the dynamics represents an out-of-steady state. In Fig. S3(a), we plot $[\![\mathcal{C}]\!]_\tau^2/\langle\mathcal{C}\rangle_\tau^2$ as a function of $(1/2)\int_0^\tau \sqrt{\mathcal{A}(t)}/t\,dt$ by circles, where the solid line shows the lower bound of Eq. (31) in the main text. Because all realizations are above the solid line, it is apparent that Eq. (31) in the main text has been numerically verified. Since the conventional thermodynamic uncertainty relation [4, 5], shown by Eq. (33) in the main text, holds when the system is in the steady state, we plot the realizations as a function of $\mathcal{A}(\tau)$ in Fig. S3(b), where the solid line now indicates $1/\mathcal{A}(\tau)$ showing the lower bound of Eq. (33) in the main text. Some data points are seen to be below the solid line, confirming that Eq. (33) in the main text does not hold for the out-of-steady state. The points below the lower bound tend to be found in the low dynamical activity region, because the initial state dependence is lost in the high dynamical activity region. Although the bound derived in Ref. [5] [Eq. (32) in the main text] is applicable to any time-independent Markov process, the denominator on the left side of Eq. (32) in the main text represents the time derivative of the average value of the observable rather than the time-integrated observable.

We also perform a similar numerical simulation for the quantum two-level atom dynamics. Again, we randomly select the model parameters (see the caption of Fig. S3(c) for the parameter ranges) and randomly generate the initial density operator $\rho(0)$ to calculate the fluctuation $[\![\mathcal{C}]\!]_\tau^2/\langle\mathcal{C}\rangle_\tau^2$. Figure S3(c) shows $[\![\mathcal{C}]\!]_\tau^2/\langle\mathcal{C}\rangle_\tau^2$ as a function of $(1/2)\int_0^\tau \sqrt{\mathcal{B}(t)}/t\,dt$ by circles, while the solid line shows $1/\tan\left[\frac{1}{2}\int_0^\tau \sqrt{\mathcal{B}(t)}/t\,dt\right]^2$. This confirms that Eq. (31) in the main text with $\mathcal{A}(t)$ replaced by $\mathcal{B}(t)$ holds for this out-of-steady state dynamics. In addition, Fig. S3(d) plots $[\![\mathcal{C}]\!]_\tau^2/\langle\mathcal{C}\rangle_\tau^2$ as a function of $\mathcal{B}(\tau)$, where the solid line denotes $1/\mathcal{B}(\tau)$. From Fig. S3(d), we confirm that the steady-state thermodynamic uncertainty relation derived in Ref. [6], which is Eq. (33) in the main text with $\mathcal{A}(\tau)$ replaced with $\mathcal{B}(\tau)$, does not hold for the out-of-steady state dynamics.

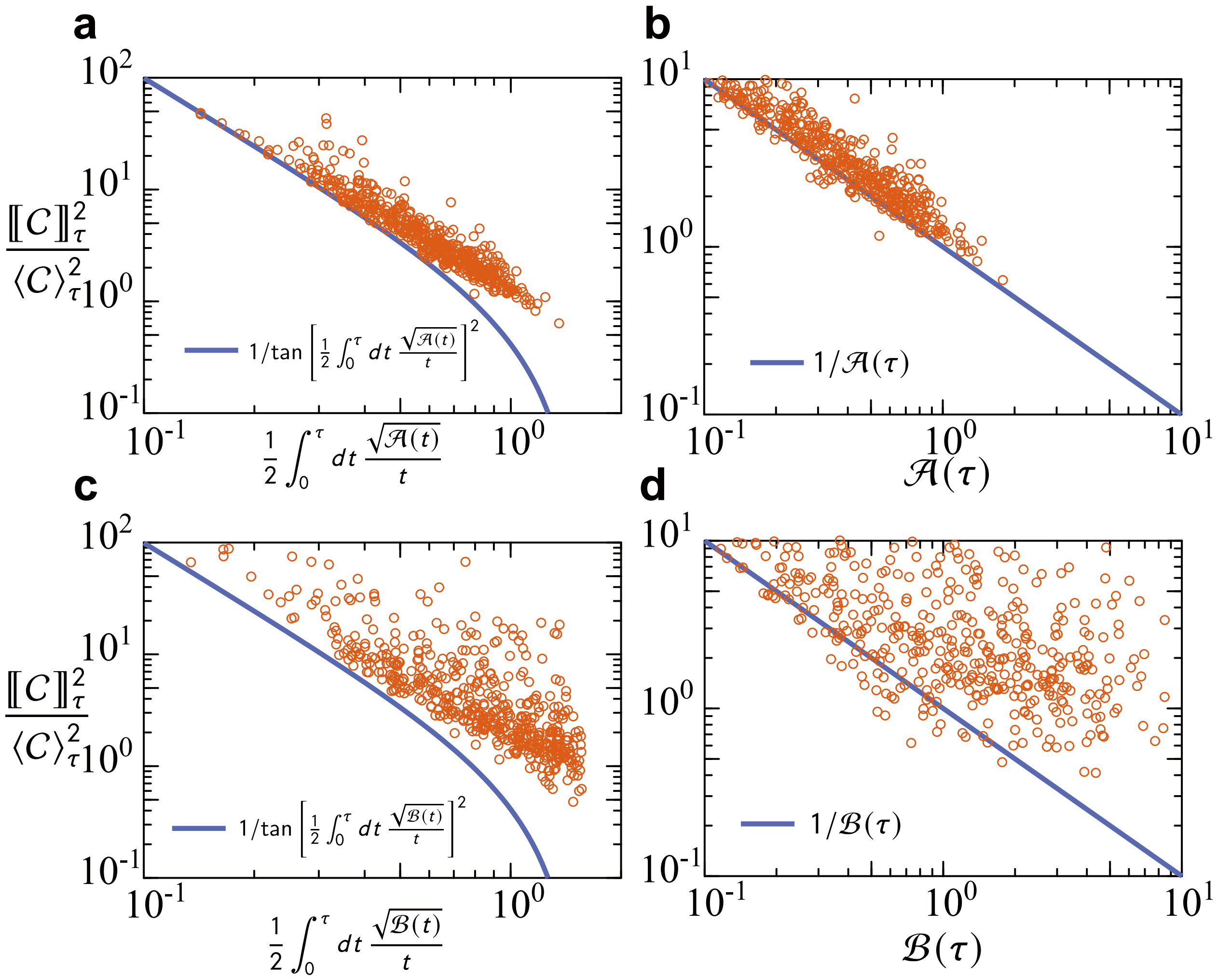

FIG. S3. **Numerical simulation of the classical and quantum thermodynamic uncertainty relations.** **a** and **b** Precision $[\![\mathcal{C}]\!]_\tau^2/\langle\mathcal{C}\rangle_\tau^2$ as a function of **a** $(1/2)\int_0^\tau \sqrt{\mathcal{A}(t)}/t\, dt$ or **b** $\mathcal{A}(\tau)$ for random realizations of the classical Markov chain. Random realizations are plotted by circles. In **a**, the solid line indicates the lower bound of Eq. (31) in the main text. In **b**, the solid line is $1/\mathcal{A}(\tau)$, which is the lower bound of the steady-state classical thermodynamic uncertainty relation [Eq. (33) in the main text]. In **a** and **b**, the parameter ranges are $\tau \in [0.1, 1]$, $W_{\mu\nu} \in [0, 1]$, and $N_S \in \{2, 3, \ldots, 5\}$. **c** and **d** Precision $[\![\mathcal{C}]\!]_\tau^2/\langle\mathcal{C}\rangle_\tau^2$ as a function of **c** $(1/2)\int_0^\tau \sqrt{\mathcal{B}(t)}/t\, dt$ or **d** $\mathcal{B}(\tau)$ for random realizations of the quantum two-level atom model. Random realizations are plotted by circles. In **c**, the solid line indicates the lower bound of Eq. (31) in the main text with $\mathcal{A}(t)$ replaced by $\mathcal{B}(t)$. In **d**, the solid line is $1/\mathcal{B}(\tau)$, which is the lower bound of the steady-state quantum thermodynamic uncertainty relation [Eq. (33) in the main text with $\mathcal{A}(\tau)$ replaced by $\mathcal{B}(\tau)$]. In **c** and **d**, the parameter ranges are $\Delta \in [0.1, 3]$, $\Omega \in [0.1, 3]$, $\kappa \in [0.1, 3]$, and $\tau \in [0.1, 1]$.

SUPPLEMENTARY NOTE 6: TIME-DEPENDENT CASE

The main paper considers a time-independent Markov process, i.e., H_{sys} and L_m are not dependent on time. Here, we consider a time-dependent case with the time-dependent operators $H_{\mathrm{sys}}(s)$ and $L_m(s)$. The time-dependent version of $\mathfrak{U}\left(\tau; (t/\tau)H_{\mathrm{sys}}, \left\{\sqrt{t/\tau}L_m\right\}\right)$ [Eq. (8) in the main text] is given by

$$\mathbb{T}\exp\left[-i\int_0^\tau ds\left(\frac{t}{\tau}H_{\mathrm{sys}}\left(\frac{t}{\tau}s\right)\otimes\mathbb{I}_{\mathrm{fld}} + \sum_m\left(i\sqrt{\frac{t}{\tau}}L_m\left(\frac{t}{\tau}s\right)\otimes\phi^\dagger(s) - i\sqrt{\frac{t}{\tau}}L_m^\dagger\left(\frac{t}{\tau}s\right)\otimes\phi(s)\right)\right)\right]. \tag{44}$$

Again, the density operator $\rho(t) = \rho_{\mathrm{sys}}^{\Psi}(t) = \mathrm{Tr}_{\mathrm{fld}}\left[|\Psi(t)\rangle\langle\Psi(t)|\right]$ yields results consistent with the time-dependent Lindblad equation. Moreover, the observable that counts the number of jump events yields the same statistics as the

genuine continuous matrix product state $|\Phi(t)\rangle$. However, note that the classical Fisher information $\mathcal{I}(t)$ does not calculated into the dynamical activity $\mathcal{A}(t)$ for the time-dependent case. The dynamical activity quantifies the extent of the dynamics of the Markov process. When the system is time-independent, the state change is solely induced by stochastic jumps. However, when the transition rate depends on time, the transition rate variation also affects the dynamics, which cannot be included in the definition of the dynamical activity. If we define the generalized dynamical activity via $\mathcal{A}_g(t) \equiv t^2\mathcal{I}(t)$ as in Eq. (38) in the main text, all the results presented in the manuscript hold for the time-dependent case. As noted in the main text, for the quantum case, all the relations hold for the time-dependent case when using the quantum dynamical activity $\mathcal{B}(\tau)$.

SUPPLEMENTARY NOTE 7: HEISENBERG UNCERTAINTY RELATION

Here, we derive the quantum thermodynamic uncertainty relation by considering the Heisenberg uncertainty relation in the bulk space. The Heisenberg uncertainty relation [7], generalized by Robertson [8], is given by

$$[\![\mathcal{X}]\!]_t[\![\mathcal{Y}]\!]_t \geq \frac{1}{2}\left|\langle[\mathcal{X},\mathcal{Y}]\rangle_t\right|, \tag{45}$$

where $\mathcal{X}$ and $\mathcal{Y}$ are arbitrary Hermitian operators, and we define the mean and standard deviation as follows:

$$\langle\mathcal{X}\rangle_t \equiv \langle\Psi(t)|\mathcal{X}|\Psi(t)\rangle, \tag{46}$$

$$[\![\mathcal{X}]\!]_t \equiv \sqrt{\langle\mathcal{X}^2\rangle_t - \langle\mathcal{X}\rangle_t^2}. \tag{47}$$

Equation (45) is a statement about the relation between the precision of two observables and their incompatibility as quantified by the commutation relation.

For notational convenience, we define

$$U(t) \equiv \mathfrak{U}\left(\tau; \frac{t}{\tau}H_{\mathrm{sys}}, \left\{\sqrt{\frac{t}{\tau}}L_m\right\}\right). \tag{48}$$

Following Ref. [9], we consider the Hermitian operator:

$$\mathcal{K}(t) \equiv -i\frac{dU^\dagger(t)}{dt}U(t). \tag{49}$$

Since $U^\dagger(t)U(t) = \mathbb{I}$, the following relation holds:

$$\frac{dU^\dagger(t)U(t)}{dt} = \frac{dU^\dagger(t)}{dt}U(t) + U^\dagger(t)\frac{dU(t)}{dt} = 0, \tag{50}$$

which guarantees the hermicity of $\mathcal{K}(t)$:

$$\mathcal{K}^\dagger(t) = iU^\dagger(t)\frac{dU(t)}{dt} = -i\frac{dU^\dagger(t)}{dt}U(t) = \mathcal{K}(t). \tag{51}$$

Let us consider a Heisenberg representation of the observable $\mathcal{X}$ considered in Eq. (45):

$$\mathcal{X}(t) = U^\dagger(t)\mathcal{X}U(t). \tag{52}$$

We substitute $\mathcal{X} \leftarrow \mathcal{X}(t)$ and $\mathcal{Y} \leftarrow \mathcal{K}(t)$ into Eq. (45) to obtain

$$\begin{aligned}[\![\mathcal{X}(t)]\!]_{t=0}[\![\mathcal{K}(t)]\!]_{t=0} &\geq \frac{1}{2}\left|\langle[\mathcal{X}(t),\mathcal{K}(t)]\rangle_{t=0}\right|, \\ &= \frac{1}{2}\left|\left\langle\frac{d\mathcal{X}(t)}{dt}\right\rangle_{t=0}\right|,\end{aligned} \tag{53}$$

where we used the Heisenberg equation for the observable $\mathcal{X}$, $\partial_t\mathcal{X}(t) = i[\mathcal{K}(t),\mathcal{X}(t)]$, when deriving the last line of Eq. (53). Apparently, $[\![\mathcal{X}(t)]\!]_{t=0} = [\![\mathcal{X}]\!]_t$ in Eq. (53). We evaluate $[\![\mathcal{K}(t)]\!]_{t=0}$ in Eq. (53) as follows:

$$[\![\mathcal{K}(t)]\!]_{t=0}^2 = \langle\mathcal{K}(t)^2\rangle_{t=0} - \langle\mathcal{K}(t)\rangle_{t=0}^2, \tag{54}$$

where

$$\begin{aligned}\langle \mathcal{K}(t)^2 \rangle_{t=0} &= \langle \mathcal{K}(t)\mathcal{K}^\dagger(t) \rangle_{t=0} \\ &= \langle \Psi(0)| \frac{dU^\dagger(t)}{dt} \frac{dU(t)}{dt} |\Psi(0)\rangle \\ &= \langle \partial_t \Psi(t) | \partial_t \Psi(t) \rangle \,, \end{aligned} \tag{55}$$

and

$$\begin{aligned}\langle \mathcal{K}(t) \rangle_{t=0} &= i \, \langle \Psi(0)| U^\dagger(t) \frac{dU(t)}{dt} |\Psi(0)\rangle \\ &= i \, \langle \Psi(t) | \partial_t \Psi(t) \rangle \,. \end{aligned} \tag{56}$$

Equations (54)–(56) show that

$$\mathcal{J}(t) = 4 [\![\mathcal{K}(t)]\!]_{t=0}^2, \tag{57}$$

where the quantum Fisher information $\mathcal{J}(t)$ is defined by Eq. (35) in the main text. Considering the observable $\mathbb{I}_{\mathrm{sys}} \otimes \mathcal{C}^\bullet$ for $\mathcal{X}$ and using Eq. (38) in the main text to convert from $\mathcal{J}(t)$ to $\mathcal{B}(t)$, we derive

$$\frac{[\![\mathcal{C}^\bullet]\!]_\tau^2}{\tau^2 \left(\partial_\tau \left\langle \mathcal{C}^\bullet \right\rangle_\tau \right)^2} \geq \frac{1}{\mathcal{B}(\tau)}, \tag{58}$$

which is the thermodynamic uncertainty relation given in Eq. (32) in the main text with $\mathcal{A}(\tau)$ replaced by $\mathcal{B}(\tau)$.

SUPPLEMENTARY NOTE 8: FUNCTION AND VARIABLE DEFINITION

In this section, we provide the variable and function definitions used in Fig. 2 in the main text. The equation numbers in the following list correspond to those in the main text.

$\mathcal{I}(t)$**:** Fisher information [Eq. (19)]

$\mathcal{J}(t)$**:** Quantum Fisher information [Eq. (35)]

Γ**:** Trajectory [Eq. (3)]

$\mathcal{P}(\Gamma, \nu, t)$**:** Probability of observing trajectory Γ and being Y_ν at time t [Eq. (17)]

$|\Psi(t)\rangle$**:** Scaled continuous matrix product state at time t [Eq. (8)]

$P(\nu, t)$**:** Probability of ν at time t [Eq. (16)]

$\rho(t)$**:** Density matrix at time t [Eq. (1)]

$\mathcal{A}(t)$**:** Dynamical activity [Eq. (23)]

$\mathcal{B}(t)$**:** Quantum dynamical activity [Eq. (38)]

$\mathcal{L}_P$**:** Bhattacharya angle [Eq. (20)]

$\mathcal{L}_D$**:** Bures angle [Eq. (36)]

$\mathcal{C}$**:** Weighted sum of the number operator $\mathcal{N}_m$ [Eqs. (13) and (26)]

$\mathcal{C}^\circ$**:** Weighted sum of the number operator $\mathcal{N}_m^\circ$ [Eqs. (14) and (29)]

$\mathcal{C}^\bullet$**:** Weighted sum of the number operator $\mathcal{N}_m^\bullet$ [Eqs. (15) and (29)]

SUPPLEMENTARY REFERENCES

[1] U. Seifert, Stochastic thermodynamics, fluctuation theorems and molecular machines, Rep. Prog. Phys. **75**, 126001 (2012).
[2] S. Gammelmark and K. Mølmer, Fisher information and the quantum Cramér-Rao sensitivity limit of continuous measurements, Phys. Rev. Lett. **112**, 170401 (2014).
[3] L. J. S. Allen, *An Introduction to Stochastic Processes with Applications to Biology*, 2nd ed. (CRC Press, 2010).
[4] J. P. Garrahan, Simple bounds on fluctuations and uncertainty relations for first-passage times of counting observables, Phys. Rev. E **95**, 032134 (2017).
[5] I. Di Terlizzi and M. Baiesi, Kinetic uncertainty relation, J. Phys. A: Math. Theor. **52**, 02LT03 (2019).
[6] Y. Hasegawa, Quantum thermodynamic uncertainty relation for continuous measurement, Phys. Rev. Lett. **125**, 050601 (2020).
[7] W. Heisenberg, Über den anschaulichen inhalt der quantentheoretischen kinematik und mechanik, Z. Phys. **43**, 172 (1927).
[8] H. P. Robertson, The uncertainty principle, Phys. Rev. **34**, 163 (1929).
[9] B. M. Escher, R. L. de Matos Filho, and L. Davidovich, General framework for estimating the ultimate precision limit in noisy quantum-enhanced metrology, Nat. Phys. **7**, 406 (2011).
